# Dye-Derived Red-Emitting Carbon Dots for Lasing and Solid-State Lighting

**DOI:** 10.1021/acsnano.3c05566

**Published:** 2023-10-23

**Authors:** Antonino Madonia, Gianluca Minervini, Angela Terracina, Ashim Pramanik, Vincenzo Martorana, Alice Sciortino, Carlo M. Carbonaro, Chiara Olla, Teresa Sibillano, Cinzia Giannini, Elisabetta Fanizza, Maria L. Curri, Annamaria Panniello, Fabrizio Messina, Marinella Striccoli

**Affiliations:** † CNR-IPCF Bari Division, Italian National Research Council, Bari, 70126, Italy; ‡ Department of Electrical and Information Engineering, Polytechnic of Bari, Bari, 70126, Italy; § Dipartimento di Fisica e Chimica “Emilio Segrè”, Università degli Studi di Palermo, Palermo 90123, Italy; ∥ Institute of Biophysics Palermo Division, Italian National Research Council, Palermo 90146, Italy; ⊥ ATeN Center, Università degli Studi di Palermo, Palermo 90123, Italy; # Department of Physics, University of Cagliari, Monserrato 09042, Italy; ∇ CNR-IC Institute of Crystallography, Italian National Research Council, Bari 70122, Italy; ○ Chemistry Department, University of Bari “Aldo Moro”, Bari 70126, Italy

**Keywords:** carbon dots, solvothermal synthesis, fluorescent nanoparticles, color converters, laser, random lasing

## Abstract

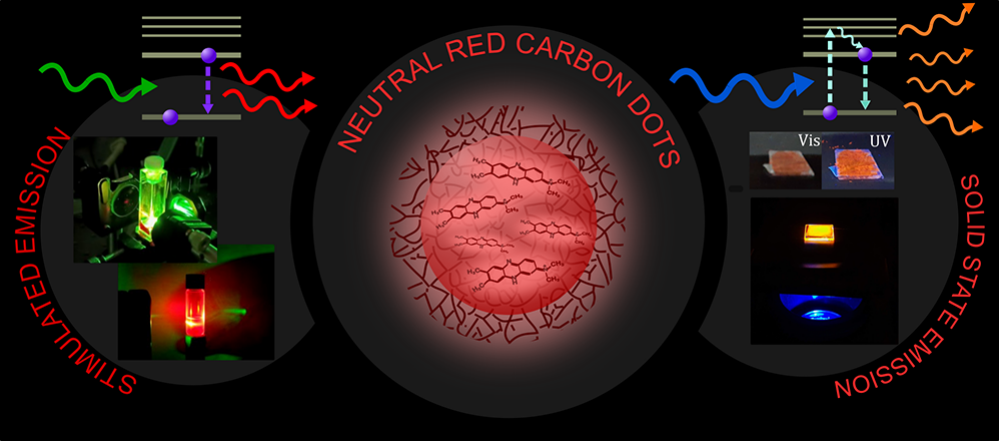

Carbon dots are carbon-based nanoparticles renowned for their intense light-emitting capabilities covering the whole visible light range. Achieving carbon dots emitting in the red region with high efficiency is extremely relevant due to their huge potential in biological applications and in optoelectronics. Currently, photoluminescence in such an energy interval is often associated with polyheterocyclic molecular domains forming during the synthesis that, however, present low emission efficiency and issues in controlling the optical features. Here, we overcome these problems by solvothermally synthesizing carbon dots starting from Neutral Red, a common red-emitting dye, as a molecular precursor. As a result of the synthesis, such molecular fluorophore is incorporated into a carbonaceous core while retaining its original optical properties. The obtained nanoparticles are highly luminescent in the red region, with a quantum yield comparable to that of the starting dye. Most importantly, the nanoparticle carbogenic matrix protects the Neutral Red molecules from photobleaching under ultraviolet excitation while preventing aggregation-induced quenching, thus allowing solid-state emission. These advantages have been exploited to develop a fluorescence-based color conversion layer by fabricating polymer-based highly concentrated solid-state carbon dot nanocomposites. Finally, the dye-based carbon dots demonstrate both stable Fabry–Perot lasing and efficient random lasing emission in the red region.

Carbon dots (CDs) are nanoparticles composed of a carbon core often smaller than 10 nm, either amorphous or crystalline,^1−5^ surrounded by an organic shell rich in chemical moieties;^6−8^ these, depending on their polarity, allow dispersion of the nanoparticles in different solvents.^9−11^ While carbon is generally regarded as an optically black material,^12^ CDs possess surprising light-emitting capabilities; their intense absorption bands can be photoexcited to express a bright fluorescence occurring mainly in the blue and green regions of the visible spectrum, often associated with high quantum yields (QYs).^13−15^ As a matter of fact, being composed of elements such as carbon, oxygen, hydrogen, and nitrogen, CDs are regarded as cost-effective fluorescent nanoparticles of easy availability.^16^ Their safe elementary composition makes them inherently nontoxic and mostly biocompatible.^17−19^ Moreover, CDs have been reported^20−24^ to strongly interact with the surrounding environment through electron or energy transfer processes that significantly influence their optical properties. During the past few years, these phenomena have been thoroughly studied to understand the mechanisms of the observed fluorescence and for the application of CDs as photosensitizers.^11^

The properties of CDs have been tested in numerous fields of application such as chemical sensing, bioimaging, nanomedicine, photocatalysis, and optoelectronics.^12,25^ As such, CDs are often regarded as ideal and sustainable alternatives to semiconductor quantum dots that are, instead, based on hazardous and/or critical materials.^16,26^ Nonetheless, many applications, mainly in optoelectronics, specifically need red-emitting CDs featuring high QYs. Yet, the full control of the CD optical properties in the red range is far from being reached,^27^ thus limiting their widespread usage. Many synthetic approaches are, in fact, often based on trial-and-error methods instead of relying on carefully planned strategies.^28^ Studies have demonstrated that red-emitting CDs display a large π–π* conjugation degree in their core.^5,14,25^ Therefore, the strategies adopted for their synthesis have frequently been based on aromatic molecules, such as phenylenediamines,^29,30^ which can react to form larger cyclic structures. In other cases, nonaromatic synthetic precursors leading to red CDs were shown to undergo cross-linking and carbonization processes, hence constructing large sp^2^ domains.^25,31^ In both cases, the red fluorescence was associated with heterocyclic molecules composed of five or more rings forming during the synthesis and incorporated into CDs.^32^ Such studies tested a wide range of precursors to obtain many different types of nanoparticles whose properties were investigated;^33^ arguably, more straightforward strategies should be found in order to overcome this time-consuming approach.

The assumption is that if the molecular fluorophores forming during the synthetic steps are finally responsible for the emission, it could be significantly more convenient to use as precursor compounds that already present such emitting properties. On this basis, during the bottom-up reaction the fluorophores would partially carbonize and contribute to the formation of carbon nanoparticles; at the same time, the noncarbonized precursor fraction would be incorporated in the structure of CDs, thus being responsible for the final optical properties of the resulting nanostructures.

Here, we pursue this strategy by using Neutral Red (NR) as a precursor. NR is an organic dye based on phenazine, often used as a staining agent,^34^ probe,^35^ and redox mediator^36^ in biological systems. Thanks to its relatively low cost and low toxicity, it has been often used in both in vitro^37^ and in vivo^38^ studies. Interestingly, its optical properties are highly sensitive to the surrounding environment and in particular to pH, being also a pH indicator (p*K*_A_ of 6.81^39^). In basic aqueous solutions, it exhibits a broad absorption band peaking at 452 nm that shifts up to 535 nm under acid conditions.^40^ Instead, its fluorescence, characterized by a signal peaking at 637 nm, has been shown not to depend on pH in water, caused by either a rapid deprotonation occurring to the acidic NR when photoexcited or interactions occurring with the solvent. In addition, NR is also significantly sensitive to environment polarity and H-bond formation.^41,42^ Then, especially in protic solvents, the NR electronic state dynamics are modified by the solvent polarity,^43^ causing a red shift of the emission band while the QY decreases. In addition, H-bonds stabilize the energy state, strengthening these effects.^42^

Uses of NR for optoelectronic applications are less common than those in the biological field, as NR emits only in solution and not from the solid state, unless there are significant modifications to its structure.^44^ In addition, while it has previously been applied as an active medium in dye lasers,^45^ its usage is not widespread, being considered less efficient with respect to other dyes, albeit both significantly less expensive and harmful. Examples of CD synthesis that use NR as precursor have already been reported in the literature,^46−48^ however without specifically studying whether and how the optical properties of the obtained nanoparticles are correlated to those of the fluorescent dye.

Here, we synthesized CDs using NR as a precursor in a solvothermal synthesis. This strategy allows carbon nanoparticles to be produced that retain the characteristics of the original fluorophore thanks to the protective action of the carbonaceous matrix. Structural and optical investigations based on NR’s sensitivity to its surroundings demonstrate that the dye molecules are confined in the CDs’ core. Therefore, the carbonaceous matrix leads to a strong photobleaching resistance of the fluorophore. Unlike its molecular counterpart, the obtained NR-based CDs emit from the solid state, proving the prevention of aggregation-induced quenching, often reported for carbon nanoparticles.^49−53^ This result makes such red-emitting nanoparticles suitable for the development of solid-state fluorescent devices as color converters for LEDs. Moreover, obtaining lasing from CDs is still challenging, especially in the red region. The few reported examples often show low stability, a very broad line shape, or lasing occurring only in the green or blue.^54^ Here, we achieve laser emission from NR-CDs in the red region since the carbonaceous structure well preserves the fluorophore optical properties. Notably, the prepared CDs are able to sustain lasing both in traditional Fabry–Pérot-like resonators, with variable geometries, and in a random lasing (RL) device, with effective performance in all of the investigated cases. In perspective, the proposed versatile preparative approach can be extended to other fluorescent dyes to produce a large variety of highly “emitting by design” CDs.

## Results and Discussion

**Figure 1:**
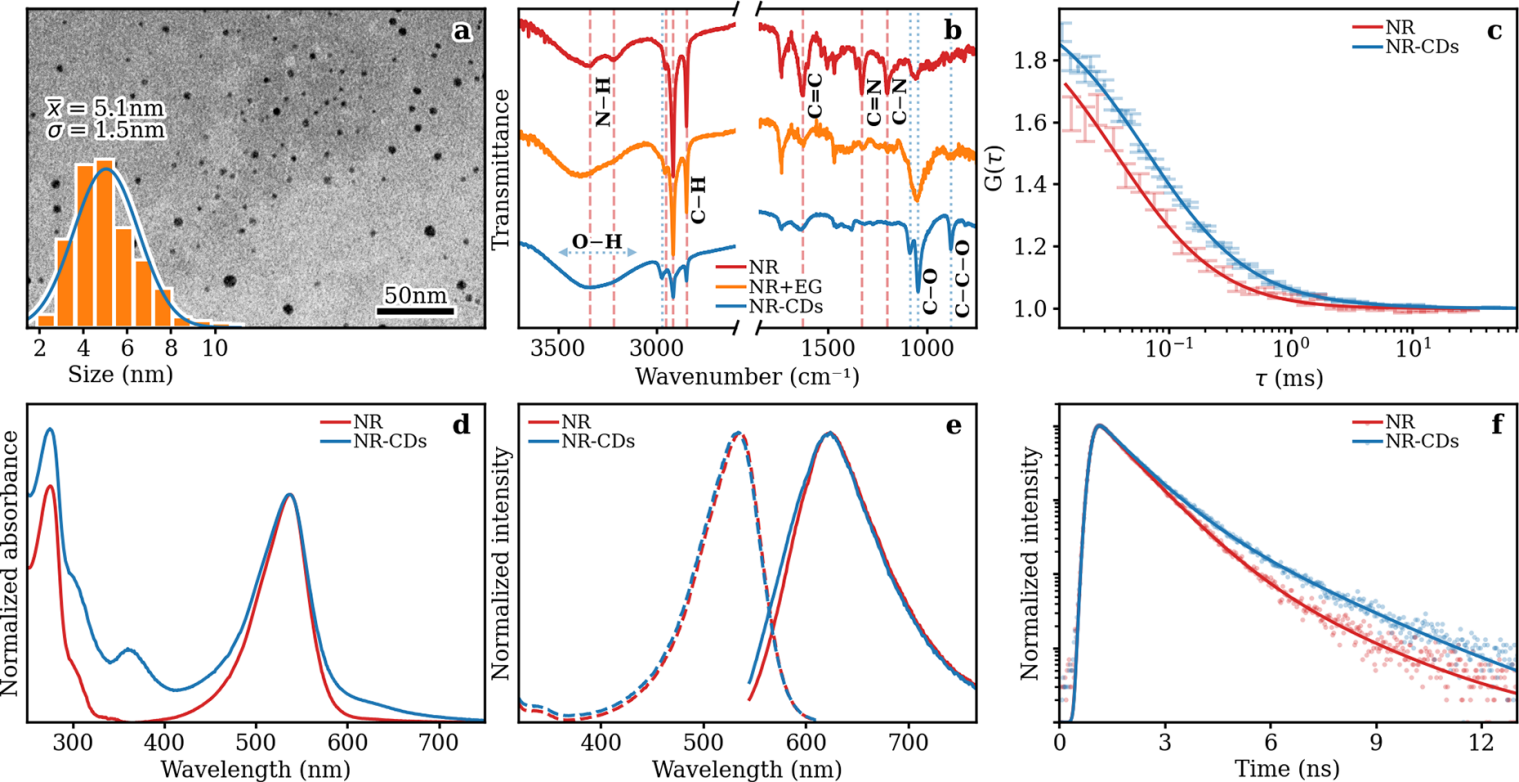
(a) TEM image of NR-CDs with the size distribution given in the inset. (b) FTIR spectra of NR-CDs, NR, and NR+EG. The identified main features are indicated by dashed lines. (c) Experimental data and fittings (continuous curves) of NR and NR-CDs FCS data with a monodisperse population of particles. Data sets are normalized by the respective autocorrelation amplitudes and averaged with a three-point rolling mean. (d) Absorbance spectra normalized on the main absorption peak. (e) Normalized PL (continuous lines, λ_exc_ = 535 nm) and PLE spectra (dashed lines, λ_em_ = 620 nm) and (f) TR emission decays (points) and relative best-fitting curves (lines) (λ_exc_ = 485 nm, λ_em_ = 620 nm) of NR-CDs and NR.

NR-CDs are synthesized in solution using NR and ethylene glycol (EG) as precursors by a solvothermal approach in an autoclave, controlling both temperature and pressure inside the reaction vessel, as described in Methods. The subsequent purification procedure allows collection of the carbonaceous nanoparticles by removing any unreacted precursor and redispersing them in ethanol (Figure S1). The purified NR-CDs have been structurally characterized by different complementary techniques. A transmission electron microscopy (TEM) investigation (Figure 1a) indicates the presence of quasi-spherical particles of 5.1 nm average diameter (σ = 1.5 nm, 29% dispersity), thus confirming the formation of CDs. The X-ray diffraction (XRD) pattern of NR-CDs (Figure S2) displays a broad band with a peak at 2θ = 26° attributed to amorphous carbon, as has often been reported for CDs in the literature.^55−57^

Fourier transform infrared spectroscopy (FTIR) spectra have been recorded to investigate the surface chemistry of NR-CDs and compared with that of the bare NR dye and with that of the precursor mixture (Figure 1b). Most of the signals of the molecular fluorophore can still be recognized in the spectrum of NR-CDs, though some differences are present (see detailed description in the Supporting Information).

The presence of the peaks in the fingerprint region observed only for the NR-CD sample suggests the formation of H-bonds after the solvothermal treatment of NR+EG, as in the spectrum of this latter sample the same signals cannot be observed as clearly. Therefore, the formation of hydrogen bonds between EG and NR can bind the molecular dye to the structure of CDs. Since TEM reveals the presence of quasi-spherical carbon nanoparticles, the formation of the NR-CD core during the synthetic process can be thought to originate from a partial carbonization of the ethylene glycol. As the thorough purification process ensures that all remaining free dye is removed from solution (Figure S1), unreacted NR molecules can then be expected to be embedded within the carbonaceous structure or linked to its surface, possibly through noncovalent interactions mediated by the organic groups present in the external shell of carbon nanoparticles.

In order to confirm that the NR fluorophore is effectively bound to the NR-CD structure, fluorescence correlation spectroscopy (FCS) experiments are performed on both the molecular dye and CD samples. Thanks to this technique it is possible to probe the average hydrodynamic radius *R*_H_ of emitting objects diffusing in solution, thus allowing a comparison of CDs and NR molecules. Indeed, the latter, based on the dynamic light scattering (DLS) data (reported in Figure S4), are expected to be smaller than the former, although DLS alone cannot disentangle fluorescent objects from any other nonemissive diffusing species. In this respect, as shown in Figure 1c, the FCS autocorrelations of the two samples, normalized by the respective amplitudes, confirm the presence of larger emitting particles in the NR-CD sample.

The fitting results presented in Table 1, in terms of the average diffusion time and the related average hydrodynamic radius, clearly indicate how, in the NR-CD sample, the emitting object has an average *R*_H_ twice as large as that of bare NR dye. This result is consistent with the occurrence of NR molecules stably associated with a carbonaceous matrix in the NR-CD sample. Therefore, in agreement with the TEM, DLS,and FTIR findings, it is possible to conclude that in NR-CDs, dye molecules are effectively connected to the structure of CDs, although it is not possible to infer if they are located on their surface or embedded in the CDs core.

**Table 1:** Diffusion Time τ and Average Hydrodynamic Radius *R*_H_ of NR-CDs and NR Obtained by Fitting the FCS Experiment Autocorrelation Curves

	τ (μs)	*R*_H_ (nm)
NR-CDs	84 ± 5	1.4 ± 0.1
NR	37 ± 5	0.8 ± 0.1

To effectively compare the optical properties between NR molecules and NR-CDs, both the molecular dye and nanoparticles are characterized by means of steady-state and time-resolved spectroscopy. The two samples share many similarities; in each sample, the ultraviolet (UV) part of the absorbance spectrum (Figure 1d) displays two bands peaking at 275 and 300 nm. An additional absorbance peak is observed only for NR-CDs at 360 nm. The relevant literature has generally associated the features in such a ectral range to either π–π* or n−π* transitions of carbonyls or connected groups^58−60^ which generally led to emission bands in the visible range. However, here the excitation of the 360 nm absorption band does not yield any inherent emission (Figure S4); therefore, we cannot conclusively associate this feature with a defined transition.

For both NR-CD and NR samples, an intense band is located at 535 nm, which is slightly broader in the case of carbon nanoparticles. The differences in the absorbance peak intensity of the two samples, especially prominent at short wavelengths, can be ascribed to scattering: due to the nature of the NR-CD dispersion, such an effect is indeed more significant for the larger carbon nanoparticles than for the NR molecules.

Excitation of the band at 535 nm generates an intense emission peak at 620 nm (Figure 1e), with QYs of 7.0 ± 0.2% and 9.3 ± 0.3%, respectively, for NR-CDs and NR. Once again, the shape of the two photoluminescence emission (PL) and excitation (PLE) spectra, yet similar, is slightly broader in the case of carbon nanoparticles (Figure 1e). Such an effect could be due to the additional inhomogeneous broadening produced by the random perturbation of the electronic transition of the NR molecules, caused by their interaction with the local environment on the surface or inside the carbonaceous core. However, the broadening is less pronounced in PLE with respect to PL spectra. Then, the contributions altering the peak shape seem to be related to processes mostly occurring from the excited state while leaving almost unaltered the ground state of the system.^61^

Finally, the decay kinetics of the red emission band is studied via time-resolved (TR) fluorescence spectroscopy. A comparison between the time decays of the two samples recorded at λ_em_ = 620 nm (λ_exc_ = 485 nm) indicates that the NR emission decay is overall faster than that of the NR-CDs, as shown in Figure 1f. In order to obtain the characteristic lifetimes, a least-squares minimization procedure is performed on the TR data, as described in the Supporting Information (Figure S6). The experimental data are fitted by a biexponential decay model, indicating that the process occurs over two different time scales. As has been extensively reported,^40^ such a biexponential behavior can be ascribed to the presence of the two different protonation states of NR that coexist in solution. The obtained best-fitting parameters, reported in Table 2, contribute to explaining the differences observed between the decay traces of NR-CDs and NR: while the τ_1_ and τ_2_ lifetime values are basically the same—within uncertainty—the relative amplitudes of these decay components are clearly different in the two samples. This evidence has two possible explanations. First, the relative amplitudes can be correlated to the different protonation states of the NR emitter. Indeed, in the NR-CDs the presence of a carbogenic matrix surrounding the NR molecules could alter their protonation equilibrium, thus explaining the observed differences. Second, the results can be also interpreted as the photoexcited state of the system behaving differently for NR and NR-CDs, due to the chemical surroundings experienced by the dye molecules, when their excited state interacts with the carbogenic matrix. Notably, the second explanation takes into account the dipolar interactions occurring between the NR excited molecules and its surroundings, finally justifying the differences in the PL shape, the variations between the relative amplitudes of the two decay components, and the decrease of the NR-CD QY in comparison to the bare NR. However, regardless of the detailed photophysical interpretation, the differences found between NR and NR-CDs are indicative of the intimate connection between the NR molecule and the CD carbonaceous structure in the NR-CD sample.

**Table 2:** Best-Fitting Parameters Obtained from the Least-Squares Minimization Procedure Performed on the Experimental TR Emission Decay Traces of NR-CDs and NR^a^

	*A*_1_ (%)	τ_1_ (ns)	*A*_2_ (%)	τ_2_ (ns)
NR-CDs	78 ± 3	0.85 ± 0.01	22 ± 3	2.1 ± 0.1
NR	92 ± 2	0.84 ± 0.02	8 ± 2	2.1 ± 0.2

a A biexponential decay model is used in the minimization procedure. Parameters *A*_i_ and τ_i_ are the relative amplitudes and the lifetimes of the fitting, respectively. Reported errors correspond to 3 times the obtained statistical error.

**Figure 2:**
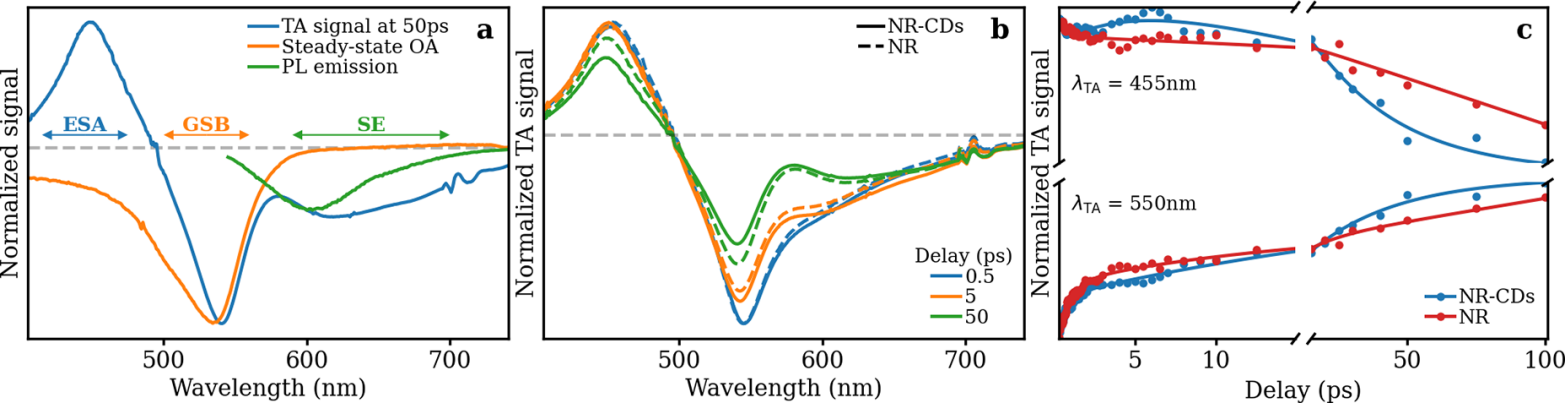
(a) TA spectra of NR-CDs recorded at 50 ps after photoexcitation (blue line). Normalized steady-state optical absorption (orange line) and PL emission (green line, λ_exc_ = 535 nm) spectra are shown for comparison, after flipping them over the *x*-axis. (b) Comparison between the TA spectra of NR-CDs (continuous curves) and NR (dashed curves) at three different delays from excitation. (c) Comparison between the TA traces of NR-CDs (blue curves) and NR (red curves) taken at the ESA and GSB signal wavelengths. The relative best-fitting curves are shown over the original data points.

To better understand the interaction between the molecular fluorophore and the surrounding carbonaceous structure, we compared the excited-state dynamics of NR-CDs and NR through ultrafast transient absorption (TA) measurements. For these measurements, NR-CDs and NR molecules are dispersed in ethanol and excited by 50 fs pulses at 535 nm. Figure S7 shows the complete set of TA spectra obtained for both NR dye and NR-CDs at variable delays from 0.12 to 150 ps after photoexcitation, while Figure 2a isolates the TA signal obtained at a 50 ps delay from photoexcitation as a representative example. All TA spectra show three contributions: (i) a negative component due to ground state bleaching (GSB) associated with the photoinduced depopulation of the ground state located around the pump wavelength, (ii) a second negative signal attributable to a strong stimulated emission (SE) at 600–670 nm, and (iii) positive signals (mostly detectable around 460 nm) due to excited-state absorption (ESA), caused by electronic transitions from the excited state toward excited states at higher energy. The position of the GSB signal reflects quite accurately the flipped-down steady-state absorption band of the sample (see Figure 2a). Most importantly, the SE signal is very pronounced and lies in the negative portion of the *y*-axis, suggesting the possibility of a significant optical gain, a necessary condition to achieve a lasing effect in the region between 600 and 700 nm. As can be seen from Figure 2b and Figure S7, the TA signal has a very similar shape in NR-CDs and NR, although some differences in the temporal evolution can be noticed (representative kinetic traces are shown in Figure 2c, along with their best fitting multiexponential curves).

In order to quantitatively compare the ultrafast dynamics of the two samples, a global least-squares fitting procedure has been performed on the first three *eigentraces* obtained by singular value decomposition (SVD) of each TA data matrix, separately for NR-CDs and NR; details are reported in the Supporting Information, and the least-squares fit results are shown in Figures S8 and S9. The four lifetimes needed to describe the ultrafast TA signal dynamics are reported in Table 3. As this fitting procedure allows obtaining the decay associated spectra (DAS)—i.e. the spectral components decaying with the same time constant—each obtained lifetime can be assigned to a specific dynamic process.

**Table 3:** Time Constants Associated to the Ultrafast Dynamics of NR-CDs and NR as Obtained by a Global Least-Squares Fitting Procedure Performed on the *Eigentraces* of the Sample TA Matrices

	τ_1_ (ps)	τ_2_ (ps)	τ_3_ (ps)
NR-CDs	0.55 ± 0.07	5.1 ± 0.6	32 ± 4
NR	0.62 ± 0.04	9.7 ± 1.1	50 ± 12

The DAS obtained for NR-CDs and NR are shown in Figures S10 and S11. In both samples, the shortest lived DAS (associated with τ_1_) describes a partial decay of the overall GSB and ESA signals through an ultrafast (<1 ps) nonradiative return to the ground state. The lifetime τ_2_ describes a shift of the GSB signal (notice the derivative-like shape in the blue region of the spectrum) along with a growth in both the ESA and SE regions. Such spectral changes can be reasonably attributed to an internal conversion, occurring within few picoseconds (see Table 3), between the initially excited state and a lower excited state responsible for the steady-state emission, as confirmed from the growth of the SE. Finally, the last DAS characterized by lifetime τ_3_ clearly indicates again an overall decay of the TA bands occurring through nonradiative processes. Notably, the τ_3_ process appears to be more pronounced, as it turns out from the relative DAS amplitudes, and faster (32 ps vs 50 ps) in NR-CDs with respect to the NR dye, thus resulting in more efficiency for the CD sample. As the associated nonradiative processes lead to the depletion of the SE, this evidence may also account for the lower NR-CDs’ QY. Finally, the last time component τ_4_ > 1 ns is associated with the long-lived signal surviving the three faster dynamics and is finally responsible for the steady-state optical properties of the system.

The TA measurements once more confirm that the spectroscopic differences between NR and NR-CDs mostly occur between the excited states, while not affecting their ground states. The global analysis and the shape of the DAS show that the processes leading to nonradiative decays are more efficient in NR-CDs compared to bare NR. Additionally, for both samples, the TA signal clearly shows an intense SE signal covering a wide interval between 600 and 670 nm. Such an SE band allows us to exploit NR-CDs as an active medium to obtain amplified stimulated emission and lasing.

The overall spectroscopic characterization indicates that the carbonaceous structure of the nanoparticles acts as a scaffold for NR molecules. The dye, besides taking part in the formation of the NR-CD core, possibly via its carbonization, serves as the sole fluorophore responsible for the observed emission.

**Figure 3:**
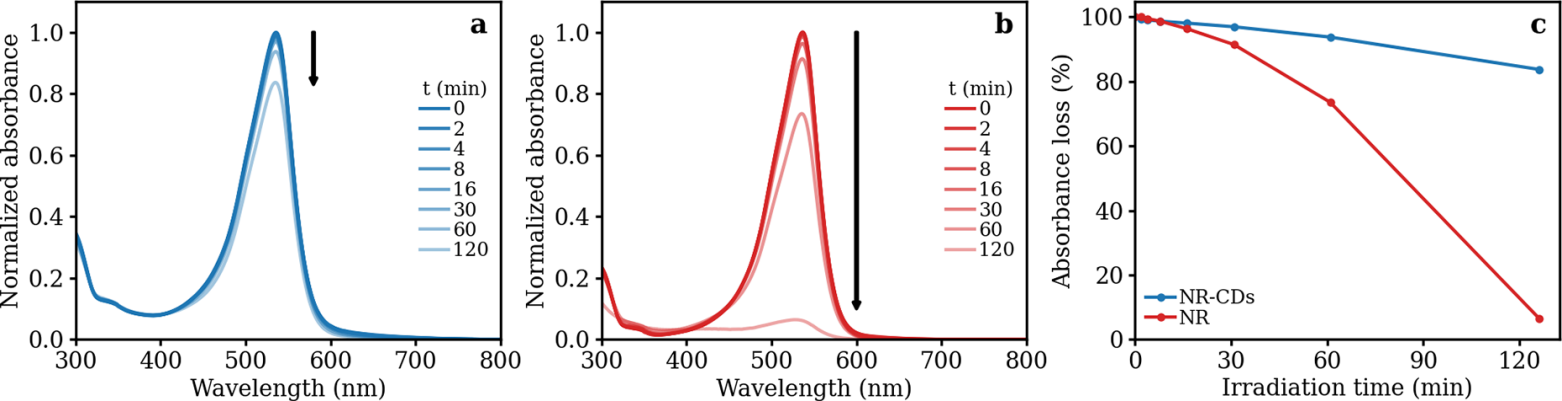
Absorbance of (a) NR-CDs and (b) NR under exposure to UV light at different irradiation times. (c) Absorbance loss over time of NR-CDs and NR upon UV irradiation.

Molecular fluorophores are very often significantly photodegraded under high-energy irradiation, thus losing their optical properties.^62^ Such a drawback is particularly detrimental for applications requiring a constant or intense irradiation of the fluorophore, such as when they are used as color-converting layers in light-emitting diodes (LEDs) or as an active medium in the resonant cavity of a dye laser. For instance, NR’s emission intensity has been shown to decrease by 50% in less than 3 h^45^ when irradiated by a Nd:YAG laser operating at 532 nm with an energy density of 32 mJ/cm^2^. Considering this aspect, the key role of the CDs’ carbonaceous matrix as a protecting scaffold for the NR fluorophore appears evident: as shown in Figure 3 and Figure S12, under UV irradiation of moderate intensity, NR-CDs display a dramatically enhanced resistance to photobleaching compared to bare NR. After 2 h of UV light exposure, the free molecular fluorophore has been almost completely degraded; the NR-CD sample, after the same irradiation time, is almost unaltered, thus pointing out the protecting role of the carbonaceous nanoparticles. Such an ability of NR-CDs to protect the fluorophores suggests that the dye molecules are mostly enclosed within their carbonaceous matrix, rather than simply anchored at the CDs’ surface. Indeed, only the presence of a matrix in the surroundings of the NR molecules could be able to shield them from oxidation following photochemical reactions that occur in the solution.

These experimental results allow us to hypothesize a formation mechanism for NR-CDs. During the initial phase of the synthesis the ethylene glycol present in the reaction batch undergoes polymerization, cross-linking, and condensation processes; similar pathways are indeed considered to commonly occur in the case of bottom-up CD synthesis.^63^ Further carbonization processes can lead to the formation of the inner CD core; due to the mild reaction temperature (200 °C), the obtained nanoparticles can be assumed to be primarily constituted by a polymeric network surrounding an amorphous carbon core.^63,64^ Such a consideration is perfectly in line with the small NR-CD size resulting from the TEM investigations and with the broad XRD peak. Based on the similarities observed between the optical properties on NR and NR-CDs, we can conclude that the mild reaction conditions prevent any degradation of the fluorescent precursor molecules. Finally, NR is embedded within the amorphous carbon matrix of CDs, as shown by both the FCS and the photobleaching experiments. As suggested by the FTIR spectra, hydrogen bonds seem to be responsible for binding the fluorescent NR together with the CD structure. Studies on the interplay between molecular fluorophores and CDs indicate that when such interaction occurs noncovalently through hydrogen bonding the optical properties of the molecular component are not significantly affected,^65^ which is in line with our results. While charge or energy transfer events may still lead to nonradiative decay pathways for the excitation, we did not observe such processes on the time scales studied through the TA experiments.

To support the assignment of the observed emission in NR-CDs and suggest a model of the structure of NR-CDs, we performed some computational chemistry simulations (details are reported in the Supporting Information). As previously reported, the main absorption band is due to the protonated form of NR typically observed in ethanol solution so that we considered two systems, the reference NR molecule and a composite made by NR and a carbonaceous structure to model the NR-CD. Accounting for the synthesis in EG, we considered the formation of a short polymeric chain of four monomer units of EG (PEG) as a model of the CD structure.^66^ Among the possibilities, the NR molecule could be linked to the CD by means of H-bonding on the amine group (NR&PEG); the optimized ground-state structure of this system displayed a H-bond of about 1.98 Å. As shown in Figure S13a, the computed UV–vis spectra and the oscillator strengths of the two model systems, NR and NR&PEG, are reported. It should be noted that the computed HOMO (highest occupied molecular orbital)–LUMO (lowest unoccupied molecular orbital) transition, the H-L gap, is blue-shifted with respect to the experimental result, as already reported in a recent computational paper.^43^ However, the main issue in the present discussion is not the correct position of the H-L gap, which could be retrieved by the proper vibronic correction,^43,67^ but the comparison of the molecular and composite systems. As expected, the optical features of the composite are very similar to those of the single NR molecule, with an almost coincident H-L gap (470 and 469 nm in the two systems, respectively), also preserving the high efficiency of the transition, with an increase of about 11% in the NR&PEG model. The MOs reported in Figure S13b clearly indicate that the transition is accomplished without the involvement of the PEG system, further supporting the proposed polymeric NR-CD model.

**Scheme 1:**
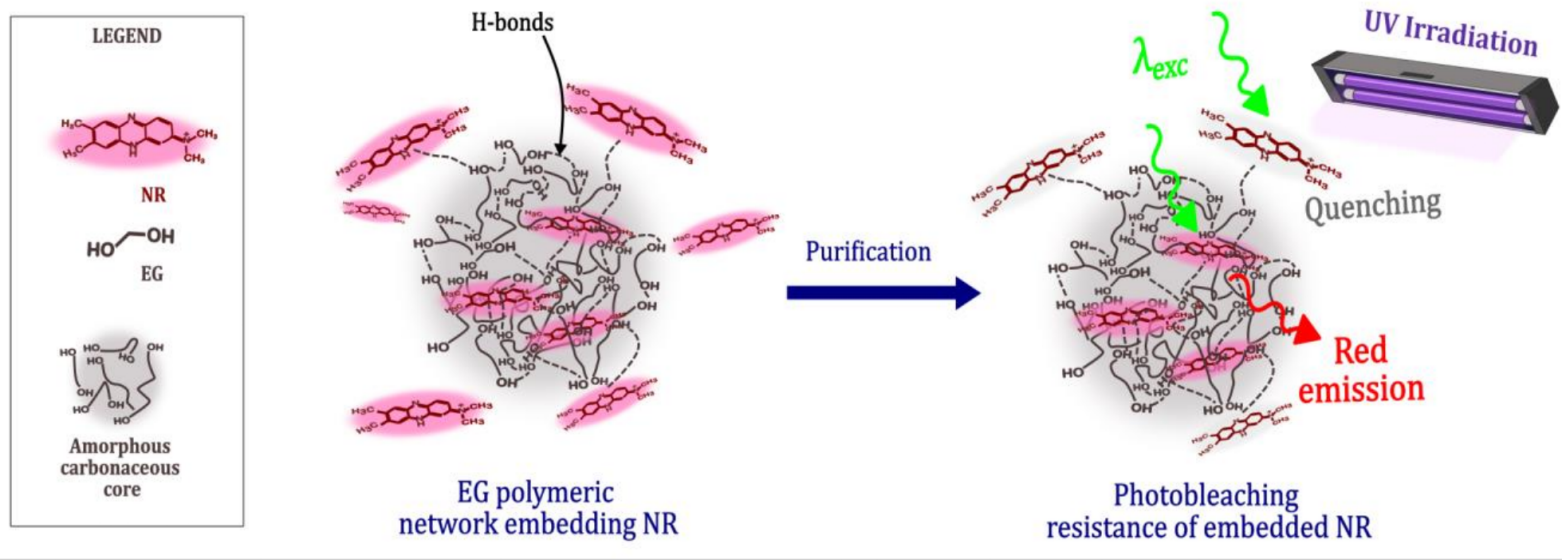
Formation, Structure, and Photobleaching Behavior of NR-CDs

Our findings, presented in Scheme 1, indicate how in NR-CDs the NR molecules are stably embedded within a polymeric carbonaceous structure that is able to protect the fluorophores from environmental degradation. As this evidence is extremely relevant in view of the usage of NR-CDs in optoelectronic devices, the possibility to exploit such an advantageous property in specific applications has been preliminarily tested.

**Figure 4:**
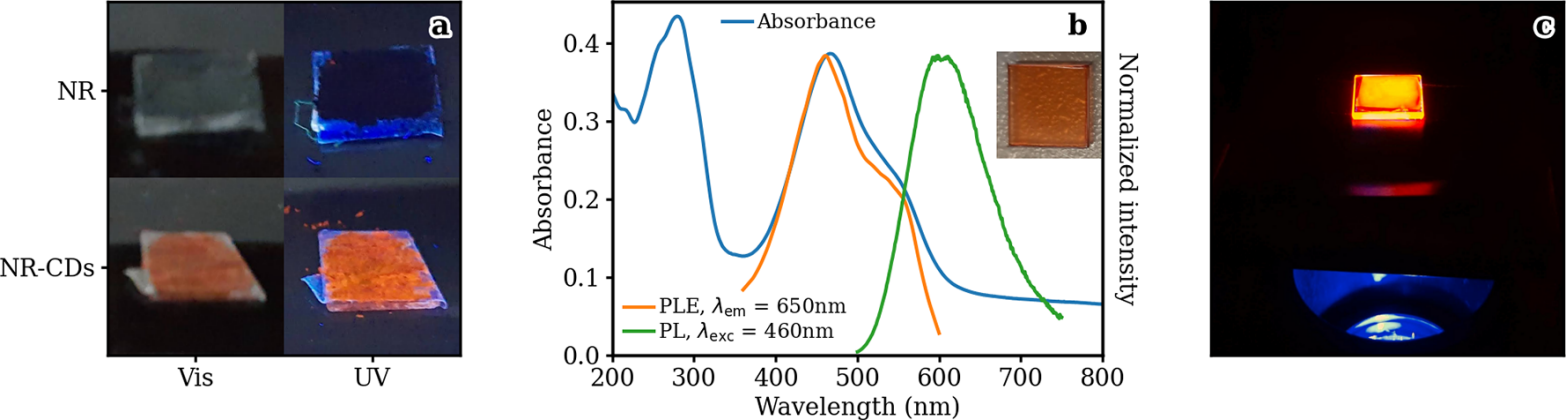
(a) Pictures of NR (top) and NR-CD (bottom) powders under visible (left) and UV (right) light. (b) Optical characterization of a NR-CDs-PVA nanocomposite spin-coated on a quartz substrate. (c) Picture of the NR-CDs-PVA nanocomposite drop-casted on a quartz substrate upon irradiation with a commercial blue InGaN LED chip.

CDs have been reported to be often affected by aggregation-induced quenching;^53^ when either in the solid state or at very high concentration in solution, their emission efficiency is significantly quenched by interparticle interactions which favor nonradiative transitions. As previously shown, the fluorophore in NR-CDs appears to be protected from the detrimental effects of UV irradiation because of the presence of the carbonaceous core; we are therefore interested in testing whether the carbonaceous matrix is also able to screen the synthesized NR-CDs from aggregation-induced effects. As shown in Figure 4a, purified NR-CD powders are surprisingly able to fluoresce in the visible region when observed under UV irradiation; in contrast, the NR dye powder appears completely dark under the same conditions. For molecular dyes, such as NR, fluorescence loss in the solid state can be attributed to a quenching caused by aggregation by π–π stacking of the dye molecules.^68^ Indeed, π–π stacking or other weak intermolecular interactions increase the probability of nonradiative recombination pathways from the excited state, thereby causing the fluorescence to be quenched. Instead, the radiative decay of excited NR fluorophores is still possible in NR-CDs thanks to the presence of the carbogenic matrix that prevents aggregation of NR molecules at the solid state.

The protective action of the carbonaceous matrix in the NR-CDs could also be profitably employed for the fabrication of light-emitting devices. Indeed, in such a context, it is often desirable to handle fluorophores at high concentration without significant loss of their spectroscopic optical properties. To further demonstrate the practical opportunities offered by the synthesized NR-CDs, a nanocomposite layer is prepared by spin-coating a concentrated solution of NR-CDs in poly(vinyl alcohol) (PVA) onto a quartz substrate, as described in Methods. Unlike the NR-CDs in solution, in the solid film the absorbance of NR-CDs-PVA displays a very large peak at 460 nm, attributed to the excited state of NR in its neutral form^40,41^ with an underlying component at around 535 nm (Figure 4b). When excited at the absorption peak wavelength, an emission band at 600 nm can be measured with an absolute QY of 2.9 ± 0.1%. Such differences with respect to NR-CDs in solution can be explained considering the modification of the chemical surroundings of the nanoparticles, moving from ethanol to the PVA matrix (Figure 1d,e). The blue shift of the PL maximum is interpreted as a widening of the HOMO–LUMO gap because of the decrease in polarity of the chemical environment within the PVA matrix.^40^ Interestingly, these results suggest that the carbogenic matrix of the NR-CDs, although able to effectively protect the NR fluorophores from photodegradative processes, does not isolate them from interactions with the external chemical environment. Notably, similar results were also reported for other CDs, characterized by emission attributed to molecular fluorophores embedded in a carbogenic matrix.^69−71^ This effect can be accounted for by considering that the reactive species acting as mediators for the photodegradative reactions can destructively interact with the external matrix before reaching the fluorophores embedded within, differently from solvent molecules or other surrounding chemical moieties.^72^

A few hundred micrometers thick film can be obtained by drop-casting a concentrated solution of NR-CDs in PVA onto a quartz substrate. As shown in Figure 4c, the nanocomposite film is able to convert the blue emission of a LED source into orange light, allowing for a proper extinction of the incident radiation through the sample volume and demonstrating the potentiality of NR-CDs to act as color converters for LEDs. Notably, the fabrication of a color-converting layer featuring both a suitable thickness and a sufficiently high fluorophore concentration can be obtained only thanks to the NR-CD resistance to aggregation-induced quenching, which allows obtaining a high-optical-density film, still preventing any significant loss in fluorescence.

**Figure 5:**
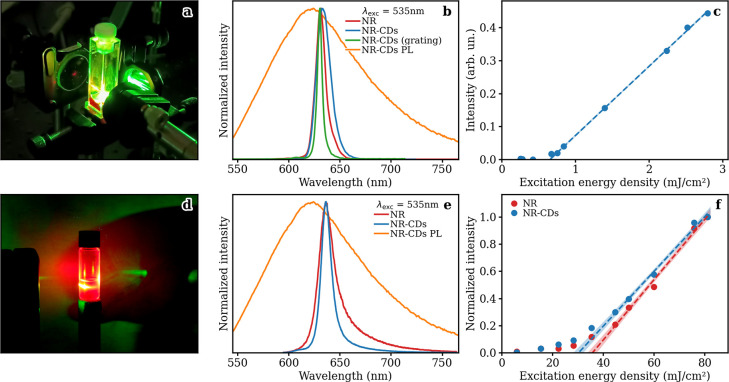
(a) Picture of Fabry–Perot red lasing emission from an NR-CD solution. (b) Stimulated emission spectra of NR dye and NR-CDs. The green curve is obtained through the substitution of one of the mirrors of the resonant cavity with a grating. (c) Output–input response curve and lasing threshold of NR-CDs. (d) Picture of red RL emission of the NR-CDs+TiO_2_ solution. (e) RL spectra of NR dye and NR-CDs. (f) Output–input response curve and lasing threshold of the two samples. The 1σ confidence interval of the threshold is shown. The data are obtained at a scatterer number density of 4.6 × 10^11^ nanoparticles/mL. The pump is at 535 nm, and the steady-state PL of NR-CDs is shown as a reference.

Laser emission in NR solutions has been partially explored for the development of dye lasers.^45^ This in principle represents a good starting point to achieve stimulated emission amplification also in NR-CDs. The possibility to obtain red lasing from the NR-CD sample is tested within a homemade resonant cavity pumped by 5 ns pulses from an external tunable laser (see Figure S14a and Methods). These experiments are conducted on a highly concentrated dispersion of NR-CDs in ethanol. As shown in Figure 5a, when exciting the NR-CDs by a focused laser beam at 535 nm with pulse energies of several tens of μJ, it was indeed possible to observe efficient lasing emission. As shown in Figure 5b, the sample emits narrow-band laser output peaking at λ_em_ = 630 nm, with a full-width at half-maximum (fwhm) of about 20 nm, which can be further reduced to 5 nm through the use of an optical grating in place of the end-cavity mirror. Lasing can be seen to be highly directional and occurs along the cavity axis, perpendicularly to the end cavity mirrors (Figure 5a).

Although it is possible to observe lasing radiation by pumping the sample between 485 and 570 nm, the lasing spectrum does not appear to be tunable. In fact, the lasing spectral position always remains very close to the maximum of SE in the TA spectra (Figure 2a), where we indeed expect the maximum gain. The best lasing efficiencies are obtained when the pump operates at wavelengths closer to the absorption maximum. The homemade cavity uses a transversal geometry with laser amplification taking place perpendicularly to the pump beam. However, it is equally possible to obtain NR-CDs lasing in a longitudinal geometry as well (see Figure S14b), where the laser pump and lasing emission have the same direction (data not shown) without significant differences in optical features.

Similar lasing properties are found for the NR dye pumped under the same conditions (Figure 5b). In order to better define the lasing characteristics of our NR-CD sample, we estimated a power yield (PY) of about 6%, calculated as the ratio between lasing emission intensity and exciting intensity, against a value of about PY = 13% found for the sample of NR dye tested at the same concentration. The output–input response curve of the stimulated emission displays a clear threshold of 0.65 mJ/cm^2^ per pulse (corresponding to 100 kW/cm^2^ taking into account the pulse duration), as can be seen in Figure 5c. Similar tests carried out on the NR dye show a threshold of 0.06 mJ/cm^2^.

We also tested the possibility of achieving RL using NR-CDs as gain media in the presence of TiO_2_ nanoparticles of about 30 nm acting as passive scatterers. As detailed in Methods, RL experiments are performed by pumping the active medium through a cylindrical lens (such as in Figure S14a) but without the use of any external mirrors providing feedback. Furthermore, the sample is contained inside a cylindrical glass vial, instead of a rectangular cuvette, so as to avoid any amplification feedback loop from the weak reflection due to the parallel walls. TiO_2_ passive scatterer nanoparticles are mixed with NR-CDs in different number densities ranging from ∼1.2 × 10^11^ to 4.6 × 10^11^ nanoparticles/mL. The resulting red RL emission generated by the sample at the highest scatterer concentration is shown in Figure 5d. It can be observed that the NR-CDs+TiO_2_ nanoparticle RL emission spans a broad angular distribution, as shown in Figure S15. Figure 5e,f shows the comparison between the RL emission characteristics of NR+TiO_2_ and NR-CDs+TiO_2_ at a numerical scatterer density of 4.6 × 10^11^ nanoparticles/mL. Both the spectral line width and RL threshold are found to be lower in the case of the NR-CDs+TiO_2_ system, respectively 12 nm compared to 19 nm and 30 mJ/cm^2^ compared to 36 mJ/cm^2^. This may be due to the participation of the NR-CDs in the RL emission process not only as gain media but also as scatterers, thanks to their structural features, thus aiding the TiO_2_ nanoparticles.^73^ As such, the NR-CD dispersion results in a more efficient RL gain medium with respect to the bare NR dye, displaying overall sharper spectral line width and lower pump intensity thresholds.

## Conclusions

Here, we demonstrate a preparative approach for CDs aimed at controlling their optical properties during the synthesis and purification steps. The use of a molecular fluorophore as precursor, namely, NR, enables the tailored engineering of red-emitting carbon-based nanoparticles, whose absorption and emission properties can be well-defined already from the sample design stage. The interplay between the fluorescent molecules and the carbonaceous matrix is verified through several characterization techniques, including structural, morphological, and optical. Photobleaching experiments prove that in such a system, NR embedded in the carbonaceous structure acts as the sole fluorophore. Such a matrix can preserve the overall optical properties of NR. A thorough comparison between the optical characteristics of the bare and enclosed fluorophore, studied down to the femtosecond scale, reveals the interaction between the NR molecules and the surrounding carbonaceous matrix.

Furthermore, enhanced photostability under prolonged irradiation and the ability of NR-CDs to display solid-state emission are key advantages of these nanoparticles. Thanks to their inherent resistance to aggregation-induced effects, NR-CDs are embedded at high concentration in PVA to produce a thick nanocomposite film working as an LED photoconversion layer. Finally, CD-based red lasing is studied both in Fabry–Pérot resonance and under RL conditions. The obtained results indicate how the scattering caused by the carbon nanoparticles, while worsening the properties of the NR-CD Fabry-Pérot resonator, aids the RL process, allowing a lowering of both its threshold and line shape width.

As a matter of principle, the presented approach could be extended to other fluorophores for the synthesis of CDs with optical properties defined *a priori* and enhanced resistance to both photobleaching and aggregation-induced quenching thanks to the protective effect of the carbon matrix. This “emission by design” preparative approach allows attaining CDs that can be effectively used as versatile and robust active components in solid-state emitting devices and in lasing applications. Additionally, our approach allows overcoming the limitation of CDs in which the intimate coupling between structural and optical properties hinders the development of nanoparticles with well-defined features. CDs with an engineered surface as well as predefined absorption and emission properties could be developed toward application in fields such as bioimaging and nanomedicine, thus allowing surmounting the trial-and-error synthetic methods which often yield carbon-based nanoparticles with undesired or unexpected final properties.

## Methods

### Chemicals

Neutral red (NR, Sigma-Aldrich, ≥90%), ethylene glycol (EG, Fisher Scientific, Certified AR for Analysis, Fisher Chemical), acetone, ethanol, hexane (Sigma-Aldrich, ACS reagent), hydrochloric acid (HCl, 37% ACS, Sigma-Aldrich), and poly(vinyl alcohol) (PVA, *M*_w_ 9000–10000, 80% hydrolyzed) were used without prior purification. TiO_2_ nanoparticles (average diameter equal to 30 nm)^74^ were purchased from Degussa (Aeroxide TiO_2_ P90) and used without any further purification.

### Synthesis of Fluorescent NR-CDs

Red-emitting fluorescent NR-CDs were prepared by modifying a procedure previously reported in the literature.^46^ Initially, 0.8 g of NR was dissolved in 20 mL of EG by stirring the mixture for 30 min at 50 °C. The solution was then poured in a 50 mL glass-lined autoclave (Series 4590 Micro Stirred Reactors, Parr Instrument Company) and heated at 200 °C. The heating apparatus allowed monitoring of the temperature reached within the reactor via a probe placed directly in contact with the reaction solution. Additionally, the solution was kept under constant agitation via a magnetically driven stirring system to ensure homogeneous heating. After 4 h of reaction the vessel was left to cool before collecting the product.

Purification of CDs was performed by multiple washing and centrifugation steps to remove the unreacted precursors. At first, acetone was added to a certain quantity of crude product in a volume ratio of 3:1. After centrifugation, the supernatant was discarded, and the same quantity of acetone was once again added to the precipitate in order to perform a second washing step. Finally, the same washing and centrifugation steps were repeated three more times by using a solution of 5% ethanol in hexane. The reaction yield after the purification, based on the mass of NR used, was equal to 62.7 ± 1.8%.

### Transmission Electron Microscopy Investigation

TEM analysis was carried out using a JEOL JEM-1400 microscope, equipped with a W filament operating at 120 kV. Micrographs were acquired using an Olympus Quemesa high-resolution CCD camera. The samples were prepared by dipping carbon-coated copper grids in diluted aqueous solutions of NR-CDs and then leaving the grids to dry under air. The size statistical analysis was performed by using free image analysis software (ImageJ, v.1.52a).

### X-ray Powder Diffraction

XRD patterns of NR-CDs powders were collected by a Rigaku RINT2500 rotating anode diffractometer (50 kV, 200 mA) equipped with a silicon strip Rigaku D/teX Ultra detector. An asymmetric Johansson Ge(111) crystal was used to select the monochromatic Cu Kα_1_ radiation (λ= 1.54056 Å). Measurements were executed in transmission mode by introducing the sample powder in a Lindemann capillary tube with a diameter of 0.5 mm. The XRD patterns were recorded in the range of 2θ = 10–120° by step scanning, using 2θ increments of 0.05° and a fixed counting time of 2/step. A qualitative analysis of the crystalline phase content was performed using the QUALX 2.0 program.^75^

### Spectroscopic Investigation

The NR-CD surface chemistry was investigated by FTIR spectroscopy using a PerkinElmer Spectrum One Fourier transform infrared spectrophotometer in an attenuated total reflection configuration. A diamond microprism of 4 mm in diameter was used as an internal reflection element. Steady-state absorbance spectra were acquired with a Cary 5000 spectrophotometer (Agilent Technologies, Inc., Santa Clara, CA, USA). PL and PLE spectra were measured with a Fluorolog 3 spectrofluorometer (HORIBA Jobin-Yvon GmbH, Bensheim, Germany) equipped with a 450 W Xe lamp as an excitation source and with double-grating excitation and emission monochromators. Absolute quantum yield (QY) values were measured using an integration sphere (Quanta-phi) internally coated with Spectralon (HORIBA Jobin Yvon GmbH, Bensheim, Germany, reflectance ≥95% between 250–2500 nm) integrated in the spectrofluorimeter.

Time-resolved fluorescence spectroscopy measurements were carried out by means of time-correlated single photon counting using a HORIBA Jobin-Yvon FluoroHub setup and a TBX photon counter as a detector. Samples were excited using a 80 ps laser diode source emitting at 485 nm (NanoLED 485L) at a repetition rate of 1 MHz; overall time resolution was measured as fwhm of the scattering signal obtained in the absence of sample and was equal to 200 ps. Steady-state absorbance, PL, PLE, QY, and TR measurements were performed by dispersing the samples in an ethanol solution to which 5% HCl aqueous solution was added at a concentration of 2.5% (v/v). The solutions to be studied were placed in quartz cuvettes with a 1 cm optical path.

### Fluorescence Correlation Spectroscopy Investigation

The FCS experiments were performed using a C9413 unit from Hamamatsu, with the excitation wavelength at λ_exc_ = 473 nm. The autocorrelation functions were computed using both the accompanying Labview software and a USB 1024-channel hardware correlator. A large number of autocorrelations obtained with short duration measurements (∼10 s) were individually recorded to allow the estimation of the error associated with each correlation point. The samples were deposited in 35 μL wells covered with a glass coverslip to avoid evaporation during the measurements.

### Absorbance Loss in Photobleaching Experiments

In absorbance loss in photobleaching experiments, the samples’ concentration (both NR and NR-CDs) was chosen so as to yield a starting absorbance of the 535 nm band equal to 1. The cuvettes containing the samples were placed under magnetic stirring and irradiated via a Hg lamp at a power density equal to 0.6 W/cm^2^ for a maximum time of 2 h. During the irradiation, the absorbance of the 535 nm band was then measured at different time intervals following the evolution for the reported exposure time.

### Preparation of Fluorescent Nanocomposites

Polymeric nanocomposite coatings based on NR-CDs were prepared employing previously reported procedures^29,76,77^ with minor variations. In detail, purified NR-CDs were dispersed in a HCl 10^–3^ M aqueous solution under vigorous stirring; subsequently PVA (0.4 g/mL) was added to the above solution, and the system was kept under magnetic stirring until an optically clear dispersion was obtained. This NR-CDs-PVA solution was spin-coated onto a quartz substrate or, to obtain a thicker layer, a NR-CDs-PVA solution (PVA 0.2 g/mL) was drop-cast onto the surface of the quartz substrate and water was allowed to slowly evaporate at 40 °C on a heating plate. To demonstrate the possibility of using them as color-converting layers, drop-casted NR-CDs nanocomposites were irradiated with a Kingbright Blue (InGaN) LED (peak wavelength: 465 nm) in a dark chamber.

### Pumping Setup and Laser Cavity

NR and NR-CD laser solutions (absorbance equal to 1.6 OD in 1 mm at the absorption peak maximum) were pumped by a tunable laser system, consisting of an optical parametric oscillator pumped by a Q-switched Nd:YAG laser, providing 5 ns pulses at 10 Hz repetition rate.

Traditional cavity-based laser experiments were conducted in two different geometries, hereafter named transversal and longitudinal (Figure S14). In the transversal geometry, the sample was contained in a 1 cm quartz cuvette, while the pumping beam was focused by a cylindrical lens (*f* = 150 mm) on a line over the front face of the cuvette, and lasing action was observed in a direction perpendicular to pumping. Spherical (*f* = 50 mm or *f* = 100 mm) or flat mirrors were positioned close to either side of the cuvette to create the optical resonator, which provided feedback for laser action. In the longitudinal geometry, the sample was contained in a cuvette with a 2 mm optical path, sandwiched between a flat metallic mirror and a dichroic mirror featuring high reflectance at the lasing wavelength and high transmittance at the pump wavelength. By means of a spherical lens (*f* = 150 mm), the pumping beam was then focused at the cuvette position through the dichroic mirror, hitting the sample at a small angle from the normal to the mirror.

For random lasing (RL) experiments, NR-CDs (absorbance at 540 nm equal to 1 OD in 1 cm) were mixed in solution with TiO_2_ nanoparticles of 30 nm in size in variable concentrations. The so-obtained active RL medium was then placed in a cylindrical glass cuvette of 13 mm outer diameter, positioned slightly away from the focal point of the lens. We used a cylindrical (in spite of square) cuvette to eliminate Fabry–Pérot cavity effects^78^ due to reflections from the cuvette walls and thus single out the effects of RL. The pump beam was sent to the RL medium through an aperture of 6 mm in diameter followed by a plano-convex cylindrical lens (*f* = 50 mm). The emission from the excited sample was collected by an optical fiber positioned ∼3 cm from it and at variable angles from the direction of the incident pump beam.

In both lasing and RL experiments, the emitted light was finally sent either to an optical fiber spectrometer (Thorlabs CCS-100) or to an intensified CCD camera (PI-MAX Princeton Instruments) for spectral analysis. In both configurations, the lasing spectra were collected and recorded on an intensified CCD camera.

### Femtosecond-Resolved Transient Absorption

The femtosecond transient absorption (TA) measurements on a solution of NR-CDs excited at 535 nm were based on a 5 kHz Ti:sapphire femtosecond amplifier (Spectra Physics Solstice-Ace) which produced 75 fs pulses peaking at 800 nm (fwhm = 30 nm) with 350 μJ/pulse energy. This beam was split in two parts by a beam splitter (80%/20%) to generate the pump and the probe, respectively. On the pump arm, pulses at 535 nm with 50 fs duration were generated in a noncollinear optical parametric amplifier pumped by the fundamental, compressed by a pair of Brewster-angle prisms, chopped at 2500 Hz, and focused on the sample by a parabolic mirror with *f* = 150 mm. On the probe arm was a white light supercontinuum (400–750 nm) focusing the 800 nm beam in a 1 mm quartz cuvette containing D_2_O. The probe was focused on the sample by the same parabolic mirror used to focus the pump. The pump–probe delay was controlled by a motorized delay stage. The probe and the pump overlapped within the sample which continuously flowed in a 200 μm thick flow cell upon the action of a peristaltic pump. After the sample, the probe beam was dispersed through a Brewster-angle silica prism and focused on the detector. The spectral resolution of this configuration was 3 nm. The temporal resolution was about 70 fs. Probe spectra were measured by a camera detector system with 1024 pixels (Glaz Linescan-I) with single-shot capability. A typical signal was obtained by averaging 5000 pumped and 5000 unpumped spectra for each delay and scanning over the pump–probe delay 10–20 times.

## Supporting Information Available

Comparison between the optical properties of NR-CDs before and after dialysis, XRD pattern of NR-CDs, details regarding the FTIR data interpretation, hydrodynamic radii of NR and NR-CDs as obtained from DLS experiments, details regarding the fluorescence correlation spectroscopy data analysis, additional PL spectra of NR and NR-CDs, time-resolved photoluminescence data analysis, additional transient absorption spectra at different delays from excitation, transient absorption data analysis, NR-CD and NR TA eigentraces and relative best-fitting curves, NR-CD and NR TA decay-associated spectra, additional data regarding the photobleaching of NR and NR-CDs, computational methods used for simulation, schemes of the homemade Fabry–Pérot cavities, and angular distribution of RL emission profile generated from NR-CDs+TiO_2_ (PDF)

## Abbreviations

CDs: carbon dots
NR: Neutral Red
QY: quantum yield
EG: ethylene glycol
TEM: transmission electron microscopy
XRD: X-ray diffraction
FTIR: Fourier transform infrared spectroscopy
FCS: fluorescence correlation spectroscopy
DLS: dynamic light scattering
UV: ultraviolet
PL: photoluminescence emission
PLE: photoluminescence excitation
TR: time-resolved
TA: transient absorption
GSB: ground-state bleaching
SE: stimulated emission
ESA: excited-state absorption
SVD: singular value decomposition
DAS: decay-associated spectra
LED: light-emitting diode
PVA: poly(vinyl alcohol)
fwhm: full-width at half-maximum
PY: power yield
RL: random lasing
