# Supplementary material for: Dye-Derived Red-Emitting Carbon Dots for Lasing and Solid-State Lighting
Source: ACS Nano. 2023 Oct 23;17(21):21274–86. doi: 10.1021/acsnano.3c05566 (PMC10655242; doi:10.1021/acsnano.3c05566)
Supplement: Supplementary file 1 — nn3c05566_si_001.pdf [file nn3c05566_si_001.pdf]

Dye-Derived Red Emitting Carbon Dots for Lasing and Solid-State Lighting – Supporting Information

Antonino Madonia[1,‡], Gianluca Minervini[1,2,‡], Angela Terracina[3], Ashim Pramanik[3], Vincenzo Martorana[4], Alice Sciortino[3,5], Carlo M. Carbonaro[6], Chiara Olla[6], Teresa Sibillano[7], Cinzia Giannini[7], Elisabetta Fanizza[1,8], Maria L. Curri[1,8], Annamaria Panniello[1,], Fabrizio Messina[3,5,*], Marinella Striccoli[1,*]*

[1] CNR-IPCF Bari Division, Italian National Research Council, Bari, 70126, Italy

[2] Department of Electrical and Information Engineering, Polytechnic of Bari, Bari, 70126, Italy

[3] Dipartimento di Fisica e Chimica "Emilio Segrè", Università degli Studi di Palermo, Palermo, 90123, Italy

[4] Institute of Biophysics Palermo Division, Italian National Research Council, Palermo, 90146, Italy

[5] ATeN Center, Università degli Studi di Palermo, Palermo, 90123, Italy

[6] Department of Physics, University of Cagliari, Monserrato, 09042, Italy

[7] CNR-IC Institute of Crystallography, Italian National Research Council, Bari, 70122, Italy

[8] Chemistry Department, University of Bari "Aldo Moro", Bari, 70126, Italy

KEYWORDS. Carbon Dots, Solvothermal Synthesis, Fluorescent Nanoparticles, Color Converters, Laser, Random Lasing.

Optical properties of dialyzed NR-CDs

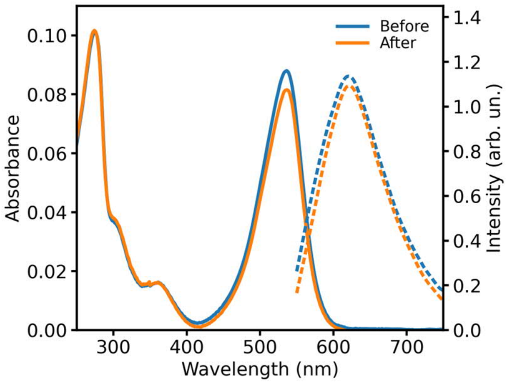

Figure S1. Absorption (continuous lines) and photoluminescence emission (dashed lines) spectra of a NR-CDs solution before and after dialysis in a membrane tube of 2 kDa nominal molecular weight cut-off.

Additional structural characterization

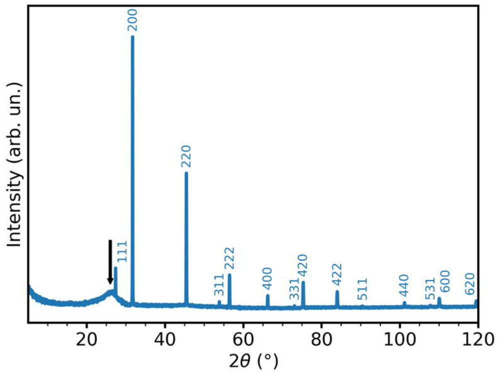

Figure S2. XRD pattern of NR-CDs; the broad feature associated to the amorphous carbonaceous core is highlighted by an arrow; additional sharp peaks are associated to the presence of NaCl contamination.

(COD CIF File http://www.crystallography.net/cod/9003308.html).

FTIR characterization

In the FTIR spectra of the NR-CDs, a broad band observed between 3600 cm^{-1} and 3000 cm^{-1} appears to be related to the stretching of O–H moieties from the EG reactant in the synthesis, which can also be observed in the NR+EG spectrum. When considering NR spectrum, instead, in the same region N–H stretching signals of amines can be observed at 3340 cm^{-1} and 3220 cm^{-1}, being these two signals reasonably present also in the nanoparticles' spectrum, though overshadowed by the intense O–H stretching band. The triplet observed at 2955 cm^{-1}, 2920 cm^{-1}, and 2850 cm^{-1} is assigned, in all the spectra, to the C–H stretching of the methyl groups present both in the dye molecule and EG. Three other prominent signals, only observed in the carbon nanoparticles' spectrum, can be also ascribed to groups related to EG. The first sharp peak, observed at 1085 cm^{-1}, is assigned to C–O stretching; furthermore, when alcohols form hydrogen bonds such signal is known to shift towards shorter frequencies thus leading to the observation of the peak found at 1045 cm^{-1}, corresponding to ~50 $kJ \cdot mol^{-1}$. Such a shift appears to have a counterpart in the O–H stretching region, as the related band displays a different shape when comparing the NR-CDs and NR+EG spectra. Nonetheless, quantitative evaluation of the peak position in this region is not reliable due to the very broad bands and to the overlapping N–H signals. Lastly, the peak at 880 cm^{-1} is a characteristic signal of alcohols arising from the stretch of the C–C–O chain. The remaining signals are all related to the NR aromatic rings stretching: those at 1630 cm^{-1}, 1330 cm^{-1}, and 1200 cm^{-1} have been previously identified in the literature[1] and are respectively assigned to the stretching vibrations of skeletal C=C, C=N, and C–N bonds. While the C=C peak can still be recognized in the all spectra, both the C=N and C–N peaks appear absent in the NR-CDs and NR+EG samples.

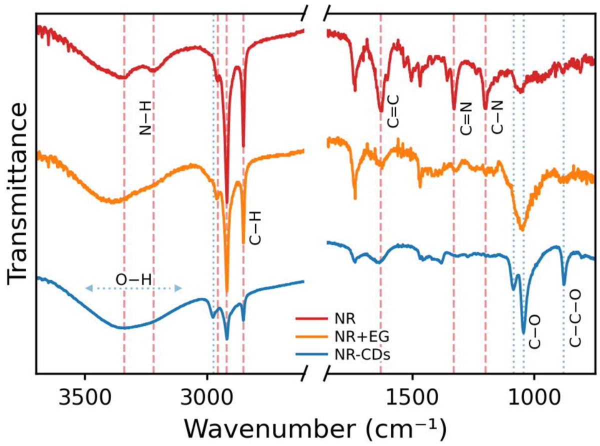

Figure S3. FTIR spectra of NR-CDs, NR, and NR+EG; the identified main features are indicated by dashed lines.

Dynamic Light Scattering measurements

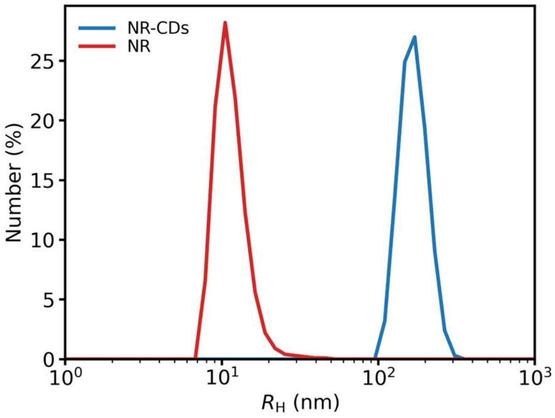

Figure S4. Comparison between the hydrodynamic radii of NR and NR-CDs as obtained from Dynamic Light Scattering experiments.

Fluorescence correlation spectroscopy data analysis

Fluorescence correlation spectroscopy data is analyzed by performing a least-squares minimization procedure. To limit the number of fitting parameters we chose to model the data with a delay time t greater than 10 µs and using a single population, using the formula:[2]

$$G(t) = A\left(1+\frac{t}{\tau}\right)^{-1}\left(1+\frac{t}{k\tau}\right)^{\frac{-1}{2}} - 1$$

where A is inversely proportional to the particle concentration, $k = 5$ is the asymmetry parameter of the point spread function, and τ is related to the hydrodynamic radius. To estimate the latter we use a 10 nM solution of Alexa-488 as a reference. We also assume that for such relatively large delay times (t > 10 µs) we can neglect the triplet state correction.

Additional optical characterization

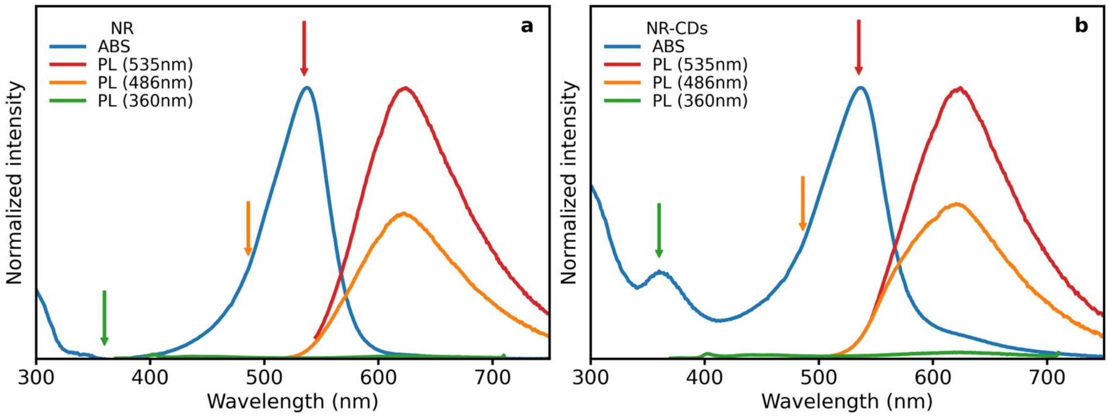

Figure S5. Comparison between the PL spectra of NR (a) and NR-CDs (b) at different excitation wavelengths (indicated in the legend). The absorbance spectra of the samples are reported for reference with arrows indicating the excitation region.

Time resolved photoluminescence data analysis.

Time resolved photoluminescence traces are analyzed by performing a least-squares minimization procedure. The following curve $I(t)$ has been used to fit the data points:

$$I(t) = f * \mathrm{IRF}(t)$$

with:

$$f(t) = \begin{cases} A_1 e^{\frac{-t-t_0}{\tau_1}} + A_2 e^{\frac{-t-t_0}{\tau_2}}, t \geq t_0 \\ 0, t < t_0 \end{cases}$$

and:

$$\mathrm{IRF}(t) = \frac{1}{\sigma\sqrt{2\pi}} e^{\frac{-(t-t_0)^2}{2\sigma^2}}$$

Here, the function $I(t)$ is the convolution between the bi-exponential decay $f(t)$ and the instrument response function IRF(t). The decay $f(t)$ is described by the amplitudes A_i that each exponential component assumes at time $t = t_0$ and by the characteristic lifetimes τ_i. The instrument response function IRF(t) is approximated by a Gaussian distribution profile centered at $t = t_0$ and width equal to σ. The results of the minimization procedure performed on the two datasets and the used IRF(t) are shown in Figure S6.

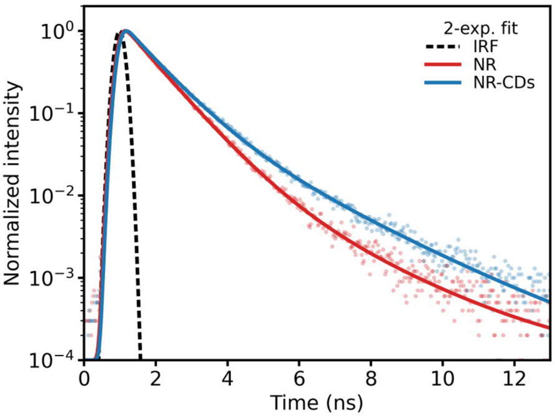

Figure S6. Least-squares fitting results of the NR and NR-CDs time-resolved emission at 620 nm (λ_{exc} = 485 nm). The Gaussian profile used as IRF is shown.

Additional transient absorption data

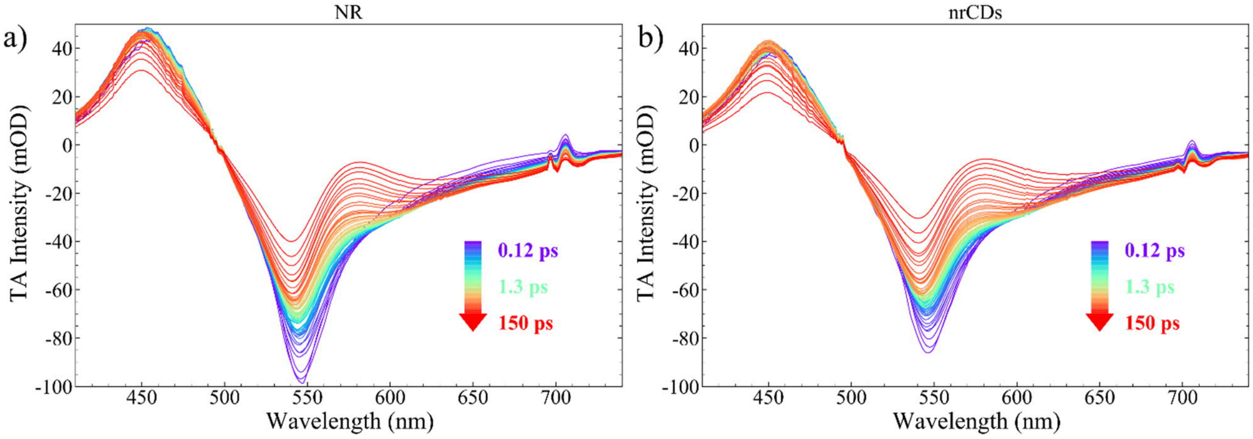

Figure S7. Transient absorption spectra of NR dye (a) and NR-CDs (b) recorded at delays ranging from 120 fs to 150 ps after photoexcitation.

Transient absorption data analysis

Transient absorption data is decomposed via the Singular Value Decomposition (SVD) algorithm:

$$\mathrm{TA}(\lambda, t)_{m,n} = \mathrm{U}(\lambda)_{m,m} \times \mathrm{S}_{m,n} \times \mathrm{V}(t)^{T}_{n,n}$$

where $\mathrm{U}(\lambda)_{m,m}$, $\mathrm{S}_{m,n}$, and $\mathrm{V}(t)_{n,n}$ are respectively the *eigenspectra*, *eigenvalues*, and *eigentraces* matrices. Such decomposition allows to quantitatively analyze the TA data by performing a global least-squares fitting procedure involving a limited number of *eigentraces* of each matrix, in order to isolate the TA signal from noise. Only the first three *eigentraces* are considered relevant to the samples' analysis, on the basis of the relative *eigenvalues*, and thus only these are included in the reported global fitting procedure. The fitting curves are described by the following function:

$$f_j(t) = \sum_{i}^{n} A_{ij}\, e^{-t/\tau_i} H_0(t) * \mathrm{IRF}(\sigma, t)$$

where A_{ij} are the pre-exponential amplitudes, τ_i the lifetimes associated to each decay, $H_0(t)$ the Heaviside step function centered at $t = 0$, and IRF(σ, t) the instrument response function supposed to be a Gaussian distribution of width σ. As the lifetimes τ_i are expected to be shared among all *eigentraces*, the least-squares fitting procedure can be performed through a global analysis. The *eigentraces* datapoints and the relative best-fitting curves are reported in Figure S8 and S9 respectively for NR-CDs and NR.

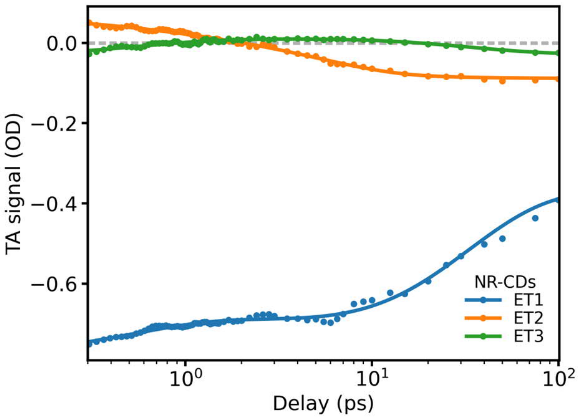

Figure S8. First three NR-CDs TA eigentraces (dots) and relative best-fitting curves (continuous curves).

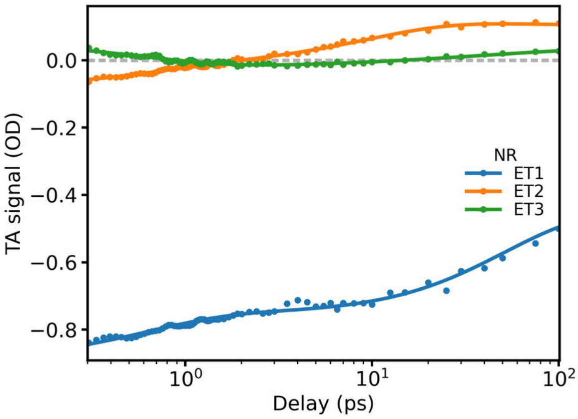

Figure S9. First three NR TA eigentraces (dots) and relative best-fitting curves (continuous curves).

From the global-least squares fit procedure performed on the *eigentraces* it is possible to obtain the Decay Associated Spectra (DAS) describing the spectral components whose kinetic is described by the same lifetime:

$$\mathrm{DAS}_i(\lambda) = \sum_j U_j\,(\lambda) s_{jj} A_{ij}$$

where U_j are the *eigenspectra*, s_{jj} are the *eigenvalues*, and A_{ij} are the pre-exponential amplitudes obtained from the fit. The obtained DAS are shown in Figure S10 and S11 respectively for NR-CDs and NR.

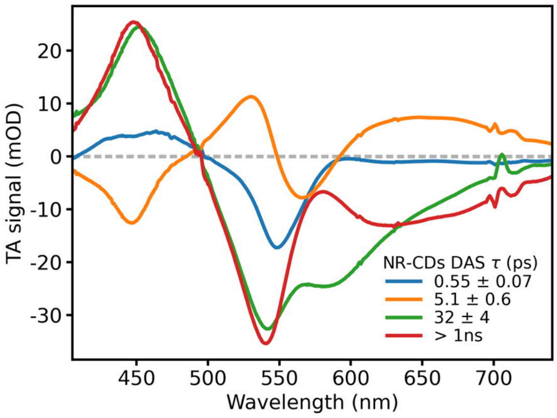

Figure 10. NR-CDs TA decay associated spectra and their associated lifetimes τ.

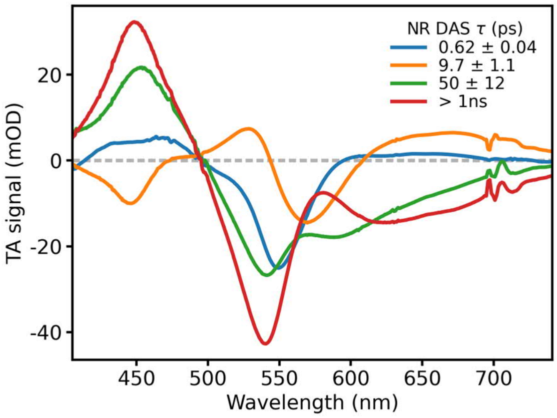

Figure S11. NR TA decay associated spectra and their associated lifetimes τ.

Additional photobleaching results

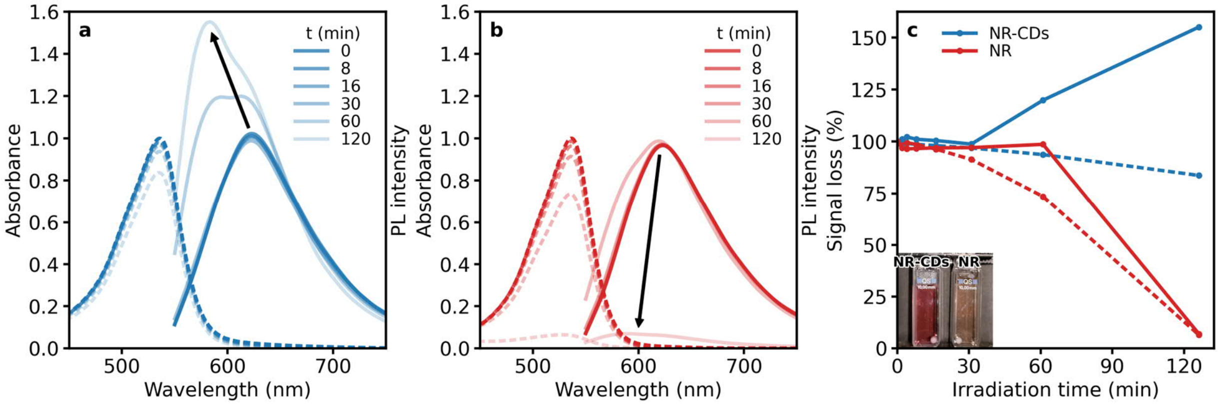

Figure S12. a, b) PL spectra (solid lines) and absorbance (dashed lines) of NR-CDs (a) and NR (b); c) PL (solid lines) and absorbance (dashed lines) intensity of NR-CDs and NR versus UV irradiation time.

Changes of PL with time under exposure to UV irradiation have been monitored. PL spectra were registered by exciting the sample's solution at 535 nm. As shown in Figure S12, after the prolonged UV light exposure NR-CDs are still able to emit when excited at 535 nm, while the NR absorption and PL features disappear almost completely due to the dye severe degradation. However, a blue-shift and a variation in the line-shape and intensity of the emission band can be observed. A detailed discussion of what happens is not straightforward. To test the photobleaching resistance, concentrated solutions were needed, so the absorbance of the used solution was approximately set to 1 at the peak maximum. This can lead to the reabsorption of emitted photons when measuring the solutions' photoluminescence, due to inner filter effects and during the photobleaching experiment, as the absorption of the sample diminishes, causing an apparent increase in PL intensity. Additionally, UV light assisted photobleaching in the protic solvent can sensitively modify the chemical environment (protonation equilibria of the species in solution, formation of ROS, local pH alterations, dilution effects), finally altering the optical properties of the fluorophore. These phenomena after 2 hour of UV irradiation cause the appearance of the

emission of the neutral form of NR ethanol solution in the yellow/orange that results more pronounced on the NR-CDs emission with respect to NR, due to the protective role of the carbonaceous matrix

Computational Methods

The structure of NR molecule was optimized with quantum mechanics calculation by means of the Gaussian 16 suite.[3] The level of theory was set within the DFT framework with both the MN12SX hybrid functional and the 6–311 ++ G(d,p) basis set.[4,5] The interaction of computed structures with solvent ethanol was accounted for by applying the Self Consistent Reaction Field (SCRF) approach and simulating the dielectric solvent through the Polarizable Continuum Model (PCM) calculation within the integral equation formalism (IEFPCM).[6] To check the optimized structures and confirm them as energy minima, we analysed the computed vibration spectra, finding no imaginary frequencies. Ball and stick representation of the structures was performed with the Gaussian package. The calculated absorbance spectra were simulated by assuming convolution of gaussian bands centred at the computed transitions, with height proportional to the oscillator strength and half width at half height of 0.333 eV. Molecular orbitals (MOs) were computed and represented by the Gaussian package, with an iso-contour value of 0.02 arb. units.

The computed HOMO-LUMO gaps are in perfect agreement with the ones reported in Kostjukov (2022).[5] As explained there the MN12SX hybrid functional is the one that allows optimal match with the experimental results once the vibronic correction is applied.[7] To mimic a polymeric CD[8] a PEG system made by the 4 monomer units of EG is considered. The optimized ground state structure displays a H-bond of 1.98 Å between the O atom of PEG and the H atom of the amine

group of NR (Figure S13). The MOs of the HOMO and LUMO transitions are depicted in the Fig. R1, showing no contribution to the transition from the PEG structure.

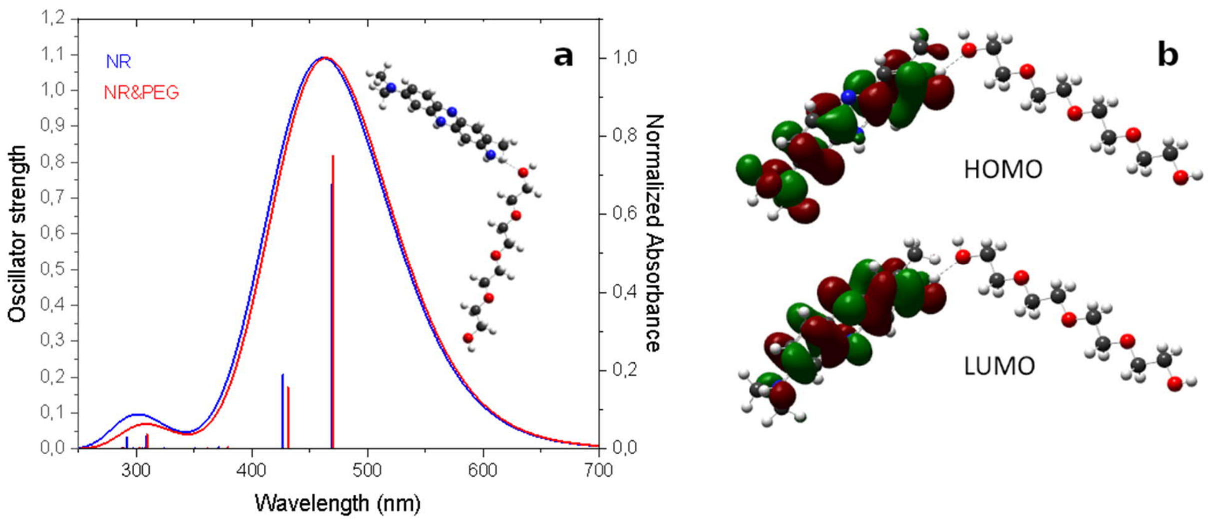

Figure S13: a) Computed oscillator strengths (vertical lines) and reconstructed UV-Vis spectra of NR and NR&PEG models b) MOs of HOMO and LUMO states for the NR&PEG system (white sphere = H atom, gray sphere = C atom, red sphere = O atom, and blue sphere = N atom). Isocontour value 0.02 a.u.

We tested also other possible structures to mimic the formation of NR-CD, such as the covalent bonding on the amine group of NR to the PEG chain (by formation of water) or the NR molecule fused on a poly-aromatic hydrocarbon system considered to model the CD network.[9] In the whole set of computed composites, the HOMO-LUMO gap did not overlap the one of the single molecule, showing a marked red-shift that increases in larger conjugated network, thus further supporting the formation of a polymeric CD.

Lasing cavity schemes

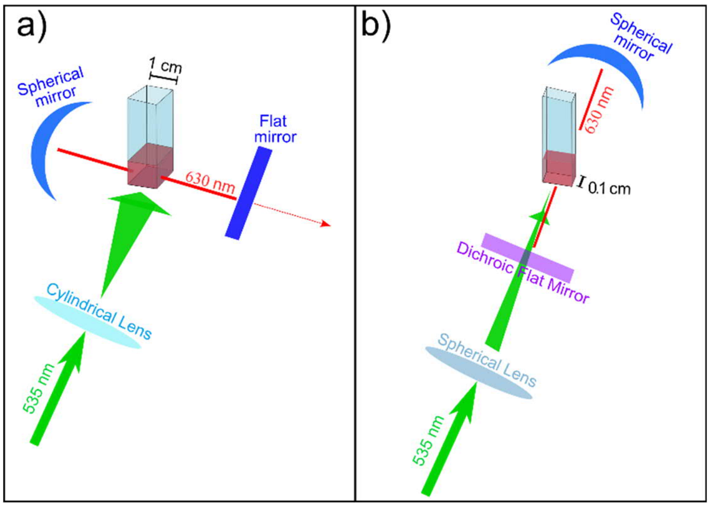

Figure S14. Schemes of the homemade Fabry-Pérot cavities built to obtain the lasing emission of the sample, using two different geometries: (a) trasversal and (b) longitudinal. The employed flat mirrors allow the output of a portion of the laser emission.

Additional lasing data

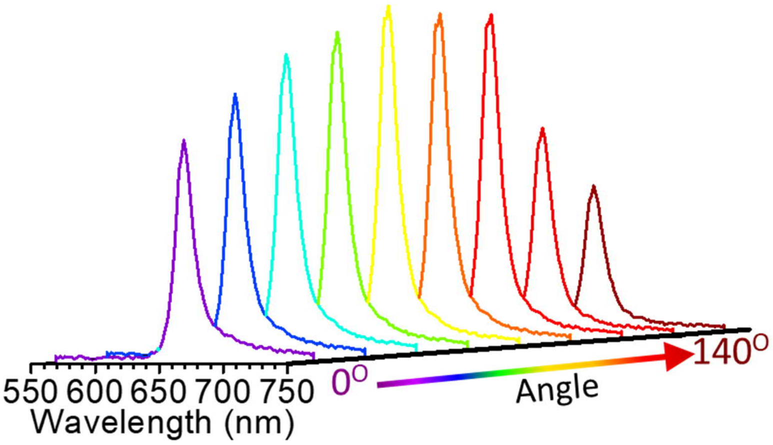

Figure S15. Angular distribution of RL emission profile generated from TiO_2+NR-CD pumped at I_p = 82 mJ/cm^2.

REFERENCES

(1) Yang, C.; Yi, J.; Tang, X.; Zhou, G.; Zeng, Y. Studies on the Spectroscopic Properties of Poly(Neutral Red) Synthesized by Electropolymerization. *React. Funct. Polym.* **2006**, *66* (11), 1336–1341. https://doi.org/10.1016/j.reactfunctpolym.2006.03.015.

(2) Lakowicz, J. R. *Principles of Fluorescence Spectroscopy*, 3rd ed.; Springer: New York, 2006.

(3) Frisch, M. J.; Trucks, G. W.; Schlegel, H. B.; Scuseria, G. E.; Robb, M. A.; Cheeseman, J. R.; Scalmani, G.; Barone, V.; Petersson, G. A.; Nakatsuji, H.; Li, X.; Caricato, M.; Marenich, A. V.; Bloino, J.; Janesko, B. G.; Gomperts, R.; Mennucci, B.; Hratchian, H. P.; Ortiz, J. V.; Izmaylov, A. F.; Sonnenberg, J. L.; Williams-Young, D.; Ding, F.; Lipparini, F.; Egidi, F.; Goings, J.; Peng, B.; Petrone, A.; Henderson, T.; Ranasinghe, D.; Zakrzewski, V. G.; Gao, J.; Rega, N.; Zheng, G.; Liang, W.; Hada, M.; Ehara, M.; Toyota, K.; Fukuda, R.; Hasegawa, J.; Ishida, M.; Nakajima, T.; Honda, Y.; Kitao, O.; Nakai, H.; Vreven, T.; Throssell, K.; Montgomery, J. A. Jr.; Peralta, J. E.; Ogliaro, F.; Bearpark, M. J.; Heyd, J. J.; Brothers, E. N.; Kudin, K. N.; Staroverov, V. N.; Keith, T. A.; Kobayashi, R.; Normand, J.; Raghavachari, K.; Rendell, A. P.; Burant, J. C.; Iyengar, S. S.; Tomasi, J.; Cossi, M.; Millam, J. M.; Klene, M.; Adamo, C.; Cammi, R.; Ochterski, J. W.; Martin, R. L.; Morokuma, K.; Farkas, O.; Foresman, J. B.; Fox, D. J. Gaussian 16, Revision C.01, 2016.

(4) Cappai, A.; Melis, C.; Stagi, L.; Ricci, P. C.; Mocci, F.; Carbonaro, C. M. Insight into the Molecular Model in Carbon Dots through Experimental and Theoretical Analysis of Citrazinic

Acid in Aqueous Solution. *J. Phys. Chem. C* **2021**, *125* (8), 4836–4845. https://doi.org/10.1021/acs.jpcc.0c10916.

(5) Kostjukov, V. V. Excitation of Neutral Red Dye in Aqueous Media: Comparative Theoretical Analysis of Neutral and Cationic Forms. *J. Mol. Model.* **2022**, *28* (4), 103. https://doi.org/10.1007/s00894-022-05098-8.

(6) Cancès, E.; Mennucci, B.; Tomasi, J. A New Integral Equation Formalism for the Polarizable Continuum Model: Theoretical Background and Applications to Isotropic and Anisotropic Dielectrics. *J. Chem. Phys.* **1997**, *107* (8), 3032–3041. https://doi.org/10.1063/1.474659.

(7) Baiardi, A.; Bloino, J.; Barone, V. General Time Dependent Approach to Vibronic Spectroscopy Including Franck–Condon, Herzberg–Teller, and Duschinsky Effects. *J. Chem. Theory Comput.* **2013**, *9* (9), 4097–4115. https://doi.org/10.1021/ct400450k.

(8) Mocci, F.; de Villiers Engelbrecht, L.; Olla, C.; Cappai, A.; Casula, M. F.; Melis, C.; Stagi, L.; Laaksonen, A.; Carbonaro, C. M. Carbon Nanodots from an In Silico Perspective. *Chem. Rev.* **2022**, *122* (16), 13709–13799. https://doi.org/10.1021/acs.chemrev.1c00864.

(9) Langer, M.; Zdražil, L.; Medveď, M.; Otyepka, M. Communication of Molecular Fluorophores with Other Photoluminescence Centres in Carbon Dots. *Nanoscale* **2023**, *15* (8), 4022–4032. https://doi.org/10.1039/D2NR05114A.
